# Supplementary material for: Extensive re-modelling of the cell wall during the development of Staphylococcus aureus bacteraemia
Source: bioRxiv. 2023 Apr 24:2023.02.23.529713. Originally published 2023 Feb 23. Preprint. [Version 2] doi: 10.1101/2023.02.23.529713 (PMC9980097; doi:10.1101/2023.02.23.529713)
Supplement: Supplement 2 [file media-2.pdf]

Strain Id	CC/ST	Infection type	Outcome30days	tcaA SNP	Accession number
ASARM61	CC22	Bacteraemia	Alive	No	ERR084492
ASARM70	CC22	Bacteraemia	Alive	No	ERR084501
ASARM71	CC22	Bacteraemia	Alive	Yes	ERR084502
ASARM72	CC22	Bacteraemia	Alive	No	ERR084503
ASARM73	CC22	Bacteraemia	Death	No	ERR084504
ASARM74	CC22	Bacteraemia	Death	No	ERR084505
ASARM77	CC22	Bacteraemia	Alive	No	ERR084506
ASARM76	CC22	Bacteraemia	Death	No	ERR084507
ASARM75	CC22	Bacteraemia	Not known	No	ERR084508
ASARM80	CC22	Bacteraemia	Alive	No	ERR084509
ASARM79	CC22	Bacteraemia	Alive	No	ERR084510
ASARM59	CC22	Bacteraemia	Alive	No	ERR084493
ASARM83	CC22	Bacteraemia	Alive	No	ERR084513
ASARM84	CC22	Bacteraemia	Not known	No	ERR084514
ASARM86	CC22	Bacteraemia	Alive	No	ERR084516
ASARM87	CC22	Bacteraemia	Alive	No	ERR084517
ASARM89	CC22	Bacteraemia	Death	No	ERR084519
ASARM62	CC22	Bacteraemia	Not known	No	ERR084494
ASARM93	CC22	Bacteraemia	Alive	No	ERR084522
ASARM95	CC22	Bacteraemia	Alive	No	ERR084523
ASARM96	CC22	Bacteraemia	Alive	No	ERR084524
ASARM97	CC22	Bacteraemia	Alive	No	ERR084525
ASARM99	CC22	Bacteraemia	Death	No	ERR084527
ASARM100	CC22	Bacteraemia	Death	No	ERR084528
ASARM101	CC22	Bacteraemia	Death	No	ERR084529
ASARM102	CC22	Bacteraemia	Death	No	ERR084530
ASARM103	CC22	Bacteraemia	Death	No	ERR084531
ASARM105	CC22	Bacteraemia	Death	No	ERR084533
ASARM107	CC22	Bacteraemia	Alive	No	ERR084534
ASARM109	CC22	Bacteraemia	Alive	No	ERR084535
ASARM114	CC22	Bacteraemia	Death	No	ERR084540
ASARM64	CC22	Bacteraemia	Alive	No	ERR109523
ASARM116	CC22	Bacteraemia	Death	Yes	ERR084541
ASARM117	CC22	Bacteraemia	Not known	No	ERR084542
ASARM118	CC22	Bacteraemia	Alive	No	ERR084543
ASARM119	CC22	Bacteraemia	Death	No	ERR084544
ASARM120	CC22	Bacteraemia	Alive	No	ERR084545
ASARM121	CC22	Bacteraemia	Alive	No	ERR084546
ASARM122	CC22	Bacteraemia	Alive	No	ERR084547
ASARM124	CC22	Bacteraemia	Alive	No	ERR084548
ASARM125	CC22	Bacteraemia	Death	No	ERR084549
ASARM126	CC22	Bacteraemia	Alive	No	ERR084550
ASARM65	CC22	Bacteraemia	Alive	No	ERR084497
ASARM127	CC22	Bacteraemia	Not known	No	ERR084551
ASARM128	CC22	Bacteraemia	Alive	No	ERR084552
ASARM129	CC22	Bacteraemia	Alive	No	
ASARM132	CC22	Bacteraemia	Alive	No	ERR084554

ASARM133	CC22	Bacteraemia	Alive	No	ERR084556
ASARM134	CC22	Bacteraemia	Death	No	ERR084557
ASARM135	CC22	Bacteraemia	Alive	No	ERR084558
ASARM136	CC22	Bacteraemia	Death	No	ERR084559
ASARM137	CC22	Bacteraemia	Death	No	ERR084560
ASARM67	CC22	Bacteraemia	Alive	No	ERR084498
ASARM138	CC22	Bacteraemia	Death	No	ERR084561
ASARM139	CC22	Bacteraemia	Alive	No	ERR084562
ASARM140	CC22	Bacteraemia	Not known	No	ERR084563
ASARM141	CC22	Bacteraemia	Not known	No	ERR084564
ASARM142	CC22	Bacteraemia	Alive	No	ERR084565
ASARM143	CC22	Bacteraemia	Alive	No	ERR084566
ASARM144	CC22	Bacteraemia	Alive	No	ERR084567
ASARM145	CC22	Bacteraemia	Not known	No	ERR084568
ASARM68	CC22	Bacteraemia	Alive	No	ERR084499
ASARM148	CC22	Bacteraemia	Not known	No	ERR084571
ASARM154	CC22	Bacteraemia	Alive	No	ERR084575
ASARM153	CC22	Bacteraemia	Death	No	ERR084576
ASARM155	CC22	Bacteraemia	Alive	No	ERR084578
ASARM160	CC22	Bacteraemia	Not known	No	ERR084579
ASARM69	CC22	Bacteraemia	Alive	No	ERR084500
ASARM162	CC22	Bacteraemia	Alive	No	ERR084581
ASARM164	CC22	Bacteraemia	Not known	No	ERR084582
ASARM163	CC22	Bacteraemia	Alive	No	ERR084583
ASARM166	CC22	Bacteraemia	Not known	No	ERR084584
ASARM165	CC22	Bacteraemia	Alive	No	ERR084585
ASARM167	CC22	Bacteraemia	Alive	No	ERR084586
ASARM168	CC22	Bacteraemia	Death	No	ERR084587
ASARM169	CC22	Bacteraemia	Alive	No	ERR084638
ASARM179	CC22	Bacteraemia	Death	No	ERR084648
ASARM181	CC22	Bacteraemia	Alive	No	ERR084650
ASARM183	CC22	Bacteraemia	Not known	No	ERR084652
ASARM184	CC22	Bacteraemia	Alive	No	ERR084653
ASARM170	CC22	Bacteraemia	Alive	No	ERR084639
ASARM191	CC22	Bacteraemia	Alive	No	ERR084658
ASARM193	CC22	Bacteraemia	Alive	No	ERR084659
ASARM196	CC22	Bacteraemia	Alive	No	ERR084661
ASARM199	CC22	Bacteraemia	Alive	No	ERR084664
ASARM200	CC22	Bacteraemia	Not known	No	ERR084665
ASARM201	CC22	Bacteraemia	Not known	No	ERR084666
ASARM171	CC22	Bacteraemia	Alive	No	ERR084640
ASARM203	CC22	Bacteraemia	Alive	No	ERR084667
ASARM204	CC22	Bacteraemia	Not known	No	ERR084668
ASARM205	CC22	Bacteraemia	Not known	No	ERR084669
ASARM208	CC22	Bacteraemia	Alive	No	ERR084671
ASARM209	CC22	Bacteraemia	Alive	No	ERR084672
ASARM207	CC22	Bacteraemia	Alive	No	ERR084673
ASARM211	CC22	Bacteraemia	Not known	No	ERR084675

ASARM212	CC22	Bacteraemia	Alive	No	ERR084676
ASARM172	CC22	Bacteraemia	Death	No	ERR084641
ASARMLT1	CC22	Bacteraemia	Alive	No	ERR084678
ASARMLT2	CC22	Bacteraemia	Alive	No	ERR084679
ASARMLT3	CC22	Bacteraemia	Alive	No	ERR084680
ASARM195	CC22	Bacteraemia	Not known	No	ERR084714
ASARM176	CC22	Bacteraemia	Alive	No	ERR084645
ASARM177	CC22	Bacteraemia	Death	No	ERR084646
ASARM217	CC22	Bacteraemia	Not known	No	ERR171907
ASARM220	CC22	Bacteraemia	Alive	No	ERR171908
ASARM221	CC22	Bacteraemia	Alive	No	ERR171909
ASARM222	CC22	Bacteraemia	Alive	No	ERR171910
ASARM223	CC22	Bacteraemia	Death	No	ERR171911
ASARM224	CC22	Bacteraemia	Alive	No	ERR171912
ASARM110	CC22	Bacteraemia	Alive	No	ERR223125
ASARM108	CC22	Bacteraemia	Alive	No	ERR223118
ASASM42	CC22	Bacteraemia	Alive	No	ERR109502
ASASM56	CC22	Bacteraemia	Death	No	ERR109515
ASASM12	CC22	Bacteraemia	Alive	No	ERR109476
ASASM61	CC22	Bacteraemia	Not known	No	ERR109520
ASASM64	CC22	Bacteraemia	Not known	No	ERR084496
ASASM71	CC22	Bacteraemia	Alive	No	ERR109528
ASASM73	CC22	Bacteraemia	Alive	Yes	ERR109530
ASASM90	CC22	Bacteraemia	Alive	Yes	ERR109540
ASASM96	CC22	Bacteraemia	Not known	No	ERR109546
ASASM97	CC22	Bacteraemia	Alive	No	ERR109547
ASASM120	CC22	Bacteraemia	Alive	Yes	ERR109567
ASASM132	CC22	Bacteraemia	Alive	No	ERR109578
ASASM138	CC22	Bacteraemia	Death	No	ERR109584
ASASM140	CC22	Bacteraemia	Alive	No	ERR109586
ASASM125	CC22	Bacteraemia	Alive	No	ERR109572
ASASM127	CC22	Bacteraemia	Not known	No	ERR109574
ASASM181	CC22	Bacteraemia	Alive	Yes	ERR109623
ASASM186	CC22	Bacteraemia	Alive	Yes	
ASASM190	CC22	Bacteraemia	Death	Yes	ERR109630
ASASM246	CC22	Bacteraemia	Alive	No	ERR114858
ASASM262	CC22	Bacteraemia	Alive	Yes	ERR114873
ASASM390	CC22	Bacteraemia	Alive	Yes	ERR172029
ASASM392	CC22	Bacteraemia	Alive	No	ERR172031
ASASM398	CC22	Bacteraemia	Alive	Yes	ERR172036
ASASM430	CC22	Bacteraemia	Alive	Yes	ERR172068
ASASM168	CC22	Bacteraemia	Not known	No	ERR223120
ASARM88	CC30	Bacteraemia	Alive	No	ERR084518
ASARM90	CC30	Bacteraemia	Not known	No	ERR084520
ASARM92	CC30	Bacteraemia	Alive	No	ERR084521
ASARM63	CC30	Bacteraemia	Alive	No	ERR084495
ASARM112	CC30	Bacteraemia	Alive	No	ERR084539
ASARM147	CC30	Bacteraemia	Death	No	

ASARM156	CC30	Bacteraemia	Not known	No	ERR084577
ASARM161	CC30	Bacteraemia	Not known	No	ERR084580
ASARM180	CC30	Bacteraemia	Not known	No	ERR084649
ASARM185	CC30	Bacteraemia	Not known	No	ERR084655
ASARM190	CC30	Bacteraemia	Alive	No	ERR084657
ASARM197	CC30	Bacteraemia	Alive	No	ERR084662
ASARM210	CC30	Bacteraemia	Not known	No	ERR084674
ASARM213	CC30	Bacteraemia	Alive	No	ERR084677
ASASM4	CC30	Bacteraemia	Death	No	ERR109472
ASASM22	CC30	Bacteraemia	Alive	No	ERR109484
ASASM35	CC30	Bacteraemia	Alive	No	ERR109495
ASASM6	CC30	Bacteraemia	Not known	No	ERR109474
ASASM43	CC30	Bacteraemia	Alive	No	ERR109503
ASASM46	CC30	Bacteraemia	Alive	No	ERR109506
ASASM47	CC30	Bacteraemia	Alive	No	ERR109507
ASASM8	CC30	Bacteraemia	Alive	No	ERR109475
ASASM59	CC30	Bacteraemia	Alive	No	ERR109518
ASASM14	CC30	Bacteraemia	Alive	No	ERR109477
ASASM17	CC30	Bacteraemia	Alive	No	ERR109480
ASASM66	CC30	Bacteraemia	Not known	No	ERR109525
ASASM67	CC30	Bacteraemia	Alive	No	ERR109526
ASASM78	CC30	Bacteraemia	Alive	No	ERR109532
ASASM79	CC30	Bacteraemia	Alive	No	ERR109533
ASASM89	CC30	Bacteraemia	Alive	No	ERR109539
ASASM98	CC30	Bacteraemia	Alive	No	ERR109548
ASASM99	CC30	Bacteraemia	Alive	No	ERR109549
ASASM100	CC30	Bacteraemia	Not known	No	ERR109550
ASASM103	CC30	Bacteraemia	Alive	No	ERR109553
ASASM116	CC30	Bacteraemia	Alive	No	ERR109563
ASASM131	CC30	Bacteraemia	Not known	Yes	ERR109577
ASASM133	CC30	Bacteraemia	Alive	Yes	ERR109579
ASASM134	CC30	Bacteraemia	Alive	No	ERR109580
ASASM147	CC30	Bacteraemia	Alive	No	ERR109593
ASASM149	CC30	Bacteraemia	Alive	No	ERR109595
ASASM156	CC30	Bacteraemia	Alive	No	ERR109601
ASASM157	CC30	Bacteraemia	Alive	No	ERR109602
ASASM159	CC30	Bacteraemia	Alive	No	ERR109604
ASASM166	CC30	Bacteraemia	Alive	No	ERR109611
ASASM126	CC30	Bacteraemia	Death	No	ERR109573
ASASM173	CC30	Bacteraemia	Alive	No	ERR109618
ASASM174	CC30	Bacteraemia	Alive	No	ERR109619
ASASM200	CC30	Bacteraemia	Alive	No	ERR109640
ASASM201	CC30	Bacteraemia	Alive	No	ERR109641
ASASM202	CC30	Bacteraemia	Alive	No	ERR109642
ASASM212	CC30	Bacteraemia	Death	No	ERR109651
ASASM220	CC30	Bacteraemia	Alive	No	ERR109658
ASASM237	CC30	Bacteraemia	Alive	No	ERR114850
ASASM250	CC30	Bacteraemia	Alive	No	ERR114862

ASASM268	CC30	Bacteraemia	Alive	No	ERR114876
ASASM272	CC30	Bacteraemia	Alive	No	ERR114880
ASASM281	CC30	Bacteraemia	Death	No	ERR114887
ASASM285	CC30	Bacteraemia	Alive	No	ERR114891
ASASM288	CC30	Bacteraemia	Alive	No	ERR114894
ASASM289	CC30	Bacteraemia	Alive	No	ERR114895
ASASM292	CC30	Bacteraemia	Not known	No	ERR114898
ASASM293	CC30	Bacteraemia	Alive	Yes	ERR114899
ASASM297	CC30	Bacteraemia	Not known	No	ERR114902
ASASM303	CC30	Bacteraemia	Alive	No	ERR114907
ASASM307	CC30	Bacteraemia	Alive	Yes	ERR114910
ASASM320	CC30	Bacteraemia	Death	No	ERR114921
ASASM329	CC30	Bacteraemia	Alive	No	ERR114928
ASASM330	CC30	Bacteraemia	Alive	No	ERR114929
ASASM331	CC30	Bacteraemia	Alive	No	ERR114930
ASASM340	CC30	Bacteraemia	Alive	No	ERR109667
ASASM345	CC30	Bacteraemia	Alive	No	ERR109672
ASASM351	CC30	Bacteraemia	Death	No	ERR109678
ASASM358	CC30	Bacteraemia	Alive	No	ERR109685
ASASM369	CC30	Bacteraemia	Not known	No	ERR109696
ASASM391	CC30	Bacteraemia	Alive	No	ERR172030
ASASM395	CC30	Bacteraemia	Not known	No	ERR172033
ASASM406	CC30	Bacteraemia	Alive	No	ERR172044
ASASM407	CC30	Bacteraemia	Alive	No	ERR172045
ASASM408	CC30	Bacteraemia	Death	No	ERR172046
ASASM436	CC30	Bacteraemia	Death	No	ERR172074
ASASM443	CC30	Bacteraemia	Alive	No	ERR172081
ASASM445	CC30	Bacteraemia	Alive	No	ERR172083
ASASM446	CC30	Bacteraemia	Death	No	ERR172084
ASASM451	CC30	Bacteraemia	Alive	No	ERR172089
ASASM457	CC30	Bacteraemia	Death	No	ERR172093
ASASM458	CC30	Bacteraemia	Alive	No	ERR172094
ASASM373	CC30	Bacteraemia	Alive	Yes	ERR223174
ASASM377	CC30	Bacteraemia	Not known	Yes	ERR223177
ASASM378	CC30	Bacteraemia	Alive	No	ERR223178
ASASM379	CC30	Bacteraemia	Alive	No	ERR223179
ASASM385	CC30	Bacteraemia	Alive	No	ERR355917
EOE003	CC30	Bacteraemia	Not known	No	ERR156428
EOE023	CC30	Bacteraemia	Not known	No	ERR156447
EOE030	CC30	Bacteraemia	Not known	No	ERR156452
EOE035	CC30	Bacteraemia	Not known	No	ERR156457
EOE041	CC30	Bacteraemia	Not known	No	ERR156462
EOE042	CC30	Bacteraemia	Not known	No	ERR156463
EOE045	CC30	Bacteraemia	Not known	No	ERR156465
EOE052	CC30	Bacteraemia	Not known	No	ERR156470
EOE054	CC30	Bacteraemia	Not known	No	ERR156472
EOE057	CC30	Bacteraemia	Not known	No	ERR156473
EOE061	CC30	Bacteraemia	Not known	No	ERR156477

EOE065	CC30	Bacteraemia	Not known	No	ERR156480
EOE072	CC30	Bacteraemia	Not known	No	ERR156486
EOE073	CC30	Bacteraemia	Not known	No	ERR156487
EOE078	CC30	Bacteraemia	Not known	No	ERR156493
EOE083	CC30	Bacteraemia	Not known	No	ERR156498
EOE084	CC30	Bacteraemia	Not known	No	ERR156499
EOE086	CC30	Bacteraemia	Not known	No	ERR156500
EOE088	CC30	Bacteraemia	Not known	No	ERR156502
EOE089	CC30	Bacteraemia	Not known	No	ERR156503
EOE090	CC30	Bacteraemia	Not known	No	ERR156504
EOE091	CC30	Bacteraemia	Not known	No	ERR156505
EOE094	CC30	Bacteraemia	Not known	No	ERR156507
EOE096	CC30	Bacteraemia	Not known	No	ERR156509
EOE097	CC30	Bacteraemia	Not known	No	ERR156510
EOE098	CC30	Bacteraemia	Not known	No	ERR156511
EOE099	CC30	Bacteraemia	Not known	No	ERR156512
EOE100	CC30	Bacteraemia	Not known	No	ERR156513
EOE101	CC30	Bacteraemia	Not known	No	ERR156514
EOE102	CC30	Bacteraemia	Not known	No	ERR156515
EOE103	CC30	Bacteraemia	Not known	No	ERR156516
EOE104	CC30	Bacteraemia	Not known	No	ERR156517
EOE105	CC30	Bacteraemia	Not known	No	ERR156518
EOE106	CC30	Bacteraemia	Not known	No	ERR156519
EOE122	CC30	Bacteraemia	Not known	No	ERR158987
EOE125	CC30	Bacteraemia	Not known	Yes	ERR158990
EOE129	CC30	Bacteraemia	Not known	No	ERR158994
EOE130	CC30	Bacteraemia	Not known	Yes	ERR158995
EOE137	CC30	Bacteraemia	Not known	No	ERR159002
EOE140	CC30	Bacteraemia	Not known	No	ERR159005
EOE154	CC30	Bacteraemia	Not known	No	ERR159018
EOE155	CC30	Bacteraemia	Not known	No	ERR159019
EOE158	CC30	Bacteraemia	Not known	No	ERR159022
EOE118	CC30	Bacteraemia	Not known	No	ERR158982
EOE198	CC30	Bacteraemia	Not known	No	ERR159048
EOE205	CC30	Bacteraemia	Not known	No	ERR159053
EOE220	CC30	Bacteraemia	Not known	No	ERR159065
EOE120	CC30	Bacteraemia	Not known	No	ERR158985
EOE225	CC30	Bacteraemia	Not known	No	ERR159070
EOE233	CC30	Bacteraemia	Not known	No	ERR177157
EOE234	CC30	Bacteraemia	Not known	No	ERR177158
EOE237	CC30	Bacteraemia	Not known	No	ERR177162
EOE268	CC30	Bacteraemia	Not known	No	ERR177193
EOE269	CC30	Bacteraemia	Not known	No	ERR177194
EOE274	CC30	Bacteraemia	Not known	No	ERR177199
EOE275	CC30	Bacteraemia	Not known	No	ERR177200
EOE276	CC30	Bacteraemia	Not known	No	ERR177201
EOE029	CC30	Bacteraemia	Not known	No	ERR177204
EOE161	CC30	Bacteraemia	Not known	No	ERR177208

EOE162	CC30	Bacteraemia	Not known	No	ERR177209
EOE163	CC30	Bacteraemia	Not known	No	ERR177211
EOE165	CC30	Bacteraemia	Not known	No	ERR177212
EOE166	CC30	Bacteraemia	Not known	No	ERR177213
EOE167	CC30	Bacteraemia	Not known	No	ERR177214
EOE169	CC30	Bacteraemia	Not known	No	ERR177215
EOE170	CC30	Bacteraemia	Not known	No	
EOE171	CC30	Bacteraemia	Not known	No	ERR177217
EOE173	CC30	Bacteraemia	Not known	No	ERR177218
EOE174	CC30	Bacteraemia	Not known	No	ERR177219
EOE175	CC30	Bacteraemia	Not known	No	ERR177220
EOE176	CC30	Bacteraemia	Not known	No	ERR177221
EOE208	CC30	Bacteraemia	Not known	No	ERR177225
EOE126	CC30	Bacteraemia	Not known	No	ERR355922
EOE229	CC30	Bacteraemia	Not known	No	ERR355923
Sa_TPS3105	ST93	Bacteraemia	Not known	No	
Sa_TPS3148	ST93	Bacteraemia	Not known	No	
Sa_TPS3161	ST93	SSTI	Not known	No	
Sa_TPS3151	ST93	SSTI	Not known	No	
Sa_TPS3165	ST93	Carriage	Not known	No	
Sa_TPS3150	ST93	Sputum	Not known	No	
Sa_TPS3171	ST93	Carriage	Not known	No	
Sa_TPS3155	ST93	Carriage	Not known	No	
Sa_TPS3118	ST93	not recorded	Not known	No	
Sa_TPS3167	ST93	ndotrachael tub	Not known	No	
Sa_TPS3162	ST93	Carriage	Not known	No	
Sa_TPS3169	ST93	SSTI	Not known	No	
Sa_TPS3106	ST93	Carriage	Not known	No	
Sa_TPS3145	ST93	SSTI	Not known	No	
Sa_TPS3158	ST93	SSTI	Not known	No	
Sa_TPS3183	ST93	not recorded	Not known	No	
Sa_TPS3134	ST93	SSTI	Not known	No	
Sa_TPS3137	ST93	SSTI	Not known	No	
Sa_TPS3181	ST93	SSTI	Not known	No	
Sa_TPS3026	ST93	Bacteraemia	Not known	No	
Sa_TPS3142	ST93	Bacteraemia	Not known	No	
Sa_TPS3164	ST93	Carriage	Not known	No	
Sa_TPS3153	ST93	Carriage	Not known	No	
Sa_TPS3104	ST93	Carriage	Not known	No	
Sa_TPS3139	ST93	SSTI	Not known	No	
Sa_TPS3149	ST93	Carriage	Not known	No	
Sa_TPS3138	ST93	SSTI	Not known	No	
Sa_TPS3160	ST93	SSTI	Not known	No	
Sa_TPS3185	ST93	Carriage	Not known	No	
Sa_TPS3176	ST93	SSTI	Not known	No	
Sa_TPS3144	ST93	SSTI	Not known	No	
Sa_TPS3174	ST93	SSTI	Not known	No	
Sa_TPS3146	ST93	SSTI	Not known	No	

Sa_TPS3133	ST93	Carriage	Not known	No	
Sa_TPS3132	ST93	not recorded	Not known	No	
Sa_TPS3152	ST93	pneumoniae	Not known	No	
Sa_TPS3135	ST93	Carriage	Not known	No	
Sa_TPS3173	ST93	Carriage	Not known	No	
Sa_TPS3140	ST93	SSTI	Not known	No	
Sa_TPS3136	ST93	Bacteraemia	Not known	No	
Sa_TPS3188	ST93	not recorded	Not known	No	
Sa_TPS3166	ST93	Carriage	Not known	No	
Sa_TPS3178	ST93	Pleural fluid	Not known	No	
Sa_TPS3157	ST93	SSTI	Not known	No	
Sa_TPS3168	ST93	SSTI	Not known	No	
Sa_TPS3189	ST93	not recorded	Not known	No	
Sa_TPS3154	ST93	SSTI	Not known	No	
Sa_TPS3163	ST93	SSTI	Not known	No	
Sa_TPS3184	ST93	Carriage	Not known	No	
Sa_TPS3182	ST93	not recorded	Not known	No	
Sa_TPS3147	ST93	SSTI	Not known	No	
Sa_TPS3177	ST93	Bacteraemia	Not known	Yes	
Sa_TPS3159	ST93	SSTI	Not known	No	
Sa_TPS3156	ST93	Carriage	Not known	No	
Sa_TPS3180	ST93	not recorded	Not known	No	
Sa_TPS3186	ST93	Carriage	Not known	No	
Sa_TPS3179	ST93	Carriage	Not known	No	
Sa_TPS3187	ST93	Carriage	Not known	No	
MR005	ST8	Bacteraemia	Not known	No	
MR007	ST8	Bacteraemia	Not known	No	
MR018	ST8	Bacteraemia	Not known	No	
MR019	ST8	Bacteraemia	Not known	No	
MR021	ST8	Bacteraemia	Not known	No	
MR022	ST8	Bacteraemia	Not known	No	
MR023	ST8	Bacteraemia	Not known	No	
MR025	ST8	Bacteraemia	Not known	No	
MR026	ST8	Bacteraemia	Not known	No	
MR027	ST8	Bacteraemia	Not known	No	
MR029	ST8	Bacteraemia	Not known	No	
MR030	ST8	Bacteraemia	Not known	No	
MR031	ST8	Bacteraemia	Not known	No	
MR035	ST8	Bacteraemia	Not known	No	
MR036	ST8	Bacteraemia	Not known	No	
MR039	ST8	Bacteraemia	Not known	No	
MR047	ST8	Bacteraemia	Not known	No	
MR051	ST8	Bacteraemia	Not known	No	
MR060	ST8	Bacteraemia	Not known	No	
MR063	ST8	Bacteraemia	Not known	No	
MR064	ST8	Bacteraemia	Not known	No	
MR065	ST8	Bacteraemia	Not known	No	
MR072	ST8	Bacteraemia	Not known	No	

MR073	ST8	Bacteraemia	Not known	No	
MR074	ST8	Bacteraemia	Not known	No	
MR077	ST8	Bacteraemia	Not known	No	
MR078	ST8	Bacteraemia	Not known	No	
MR081	ST8	Bacteraemia	Not known	No	
MR083	ST8	Bacteraemia	Not known	No	
MR084	ST8	Bacteraemia	Not known	No	
MR087	ST8	Bacteraemia	Not known	No	
MR090	ST8	Bacteraemia	Not known	No	
MR091	ST8	Bacteraemia	Not known	No	
MR096	ST8	Bacteraemia	Not known	No	
MR107	ST8	Bacteraemia	Not known	No	
MR110	ST8	Bacteraemia	Not known	No	
USFL008	ST8	Carriage	Not known	No	
USFL009	ST8	Carriage	Not known	No	
USFL012	ST8	Carriage	Not known	Yes	
USFL028	ST8	Carriage	Not known	No	
USFL042	ST8	Carriage	Not known	No	
USFL061	ST8	Carriage	Not known	No	
USFL063	ST8	Carriage	Not known	No	
USFL074	ST8	Carriage	Not known	No	
USFL077	ST8	Carriage	Not known	No	
USFL082	ST8	Carriage	Not known	No	
USFL093	ST8	Carriage	Not known	No	
USFL119	ST8	Carriage	Not known	No	
USFL130	ST8	Carriage	Not known	No	
USFL141	ST8	Carriage	Not known	No	
USFL153	ST8	Carriage	Not known	No	
USFL156	ST8	Carriage	Not known	No	
USFL166	ST8	Carriage	Not known	No	
USFL167	ST8	Carriage	Not known	No	
USFL169	ST8	Carriage	Not known	No	
USFL182	ST8	Carriage	Not known	No	
USFL200	ST8	Carriage	Not known	No	
USFL211	ST8	Carriage	Not known	No	
USFL213	ST8	Carriage	Not known	No	
USFL224	ST8	Carriage	Not known	No	
USFL225	ST8	Carriage	Not known	No	
USFL230	ST8	Carriage	Not known	No	
USFL231	ST8	Carriage	Not known	No	
USFL243	ST8	Carriage	Not known	No	
USFL248	ST8	Carriage	Not known	No	
USFL259	ST8	Carriage	Not known	No	
USFL263	ST8	Carriage	Not known	No	
USFL267	ST8	Carriage	Not known	No	
USFL269	ST8	Carriage	Not known	No	
USFL271	ST8	Carriage	Not known	No	
USFL272	ST8	Carriage	Not known	No	

USFL302	ST8	Carriage	Not known	No	
USFL303	ST8	Carriage	Not known	No	
USFL304	ST8	Carriage	Not known	No	
USFL016	ST8	SSTI	Not known	No	
USFL018	ST8	SSTI	Not known	No	
USFL020	ST8	SSTI	Not known	No	
USFL021	ST8	SSTI	Not known	Yes	
USFL034	ST8	SSTI	Not known	No	
USFL035	ST8	SSTI	Not known	No	
USFL036	ST8	SSTI	Not known	No	
USFL039	ST8	SSTI	Not known	No	
USFL056	ST8	SSTI	Not known	No	
USFL057	ST8	SSTI	Not known	No	
USFL059	ST8	SSTI	Not known	No	
USFL069	ST8	SSTI	Not known	No	
USFL095	ST8	SSTI	Not known	No	
USFL097	ST8	SSTI	Not known	No	
USFL103	ST8	SSTI	Not known	No	
USFL110	ST8	SSTI	Not known	No	
USFL111	ST8	SSTI	Not known	No	
USFL113	ST8	SSTI	Not known	No	
USFL136	ST8	SSTI	Not known	No	
USFL137	ST8	SSTI	Not known	No	
USFL138	ST8	SSTI	Not known	No	
USFL139	ST8	SSTI	Not known	No	
USFL149	ST8	SSTI	Not known	No	
USFL152	ST8	SSTI	Not known	No	
USFL158	ST8	SSTI	Not known	No	
USFL159	ST8	SSTI	Not known	No	
USFL160	ST8	SSTI	Not known	No	
USFL162	ST8	SSTI	Not known	No	
USFL164	ST8	SSTI	Not known	No	
USFL165	ST8	SSTI	Not known	No	
USFL173	ST8	SSTI	Not known	No	
USFL174	ST8	SSTI	Not known	No	
USFL194	ST8	SSTI	Not known	No	
USFL198	ST8	SSTI	Not known	No	
USFL218	ST8	SSTI	Not known	No	
USFL219	ST8	SSTI	Not known	No	
USFL220	ST8	SSTI	Not known	No	
USFL221	ST8	SSTI	Not known	No	
USFL222	ST8	SSTI	Not known	No	
USFL223	ST8	SSTI	Not known	No	
USFL237	ST8	SSTI	Not known	No	
USFL239	ST8	SSTI	Not known	No	
USFL240	ST8	SSTI	Not known	No	
USFL250	ST8	SSTI	Not known	No	
USFL255	ST8	SSTI	Not known	No	

USFL256	ST8	SSTI	Not known	No	
USFL258	ST8	SSTI	Not known	No	
USFL273	ST8	SSTI	Not known	No	
USFL274	ST8	SSTI	Not known	No	
USFL276	ST8	SSTI	Not known	No	
USFL277	ST8	SSTI	Not known	No	
USFL279	ST8	SSTI	Not known	No	
USFL282	ST8	SSTI	Not known	No	
USFL319	ST8	SSTI	Not known	No	
USFL320	ST8	SSTI	Not known	No	
USFL326	ST8	SSTI	Not known	No	
USFL327	ST8	SSTI	Not known	No	
USFL330	ST8	SSTI	Not known	No	
USFL339	ST8	SSTI	Not known	No	
USFL341	ST8	SSTI	Not known	No	
